# The Change in HbA1c Associated with Initial Adherence and Subsequent Change in Adherence among Diabetes Patients Newly Initiating Metformin Therapy

**DOI:** 10.1155/2016/9687815

**Published:** 2016-08-07

**Authors:** Gregory A. Nichols, A. Gabriela Rosales, Teresa M. Kimes, Kaan Tunceli, Karen Kurtyka, Panagiotis Mavros

**Affiliations:** ^1^Kaiser Permanente Center for Health Research, Portland, OR 97227, USA; ^2^Merck & Co., Inc., Kenilworth, NJ 07033, USA

## Abstract

*Introduction*. Whether* changes* in adherence are associated with* changes* in HbA1c is assumed but not known.* Methods*. We conducted a observational study of 2,844 type 2 diabetes patients who initiated metformin as their first antihyperglycemic drug. Using HbA1c measures before, 6–12 months after, and up to 3 years after metformin initiation, we analyzed HbA1c change as a function of initial adherence and change in adherence.* Results*. Compared with no adherence, initial adherence of 50–79% was associated with an adjusted reduction in HbA1c of 0.45% while adherence ≥80% was associated with HbA1c reduction of 0.73%. Change from some initial adherence (1–79%) to total nonadherence was associated with 0.25% increase in HbA1c. Change from some to full adherence was associated with an HbA1c decrease of 0.15%. Those associations were accentuated among patients not in glycemic control: change from some to no adherence was associated with an HbA1c increase of 0.63% and change from some to full adherence was associated with an HbA1c decrease of 0.40%.* Conclusions*. Initial adherence to newly prescribed metformin therapy produces substantial HbA1c reduction. Among those with modest adherence but suboptimal glycemic control, the difference between moving to full adherence versus nonadherence results in lower HbA1c of one percentage point.

## 1. Introduction

The American Diabetes Association (ADA) recommends initiation of metformin at the time of or soon after diagnosis of type 2 diabetes [1]. Research supports this recommendation; metformin is most effective when initiated early in the course of diabetes and while A1C levels are still low [2, 3]. Regardless of when it is initiated, the effectiveness of any pharmacotherapy requires that the patients adhere to their treatment regimen. Surprisingly, however, most previous studies of the impact of adherence on glycemic control report small effects, with every 10% increase in adherence associated with a decrease of 0.1% to 0.3% in A1C [4]. This is due, in part, to the way adherence is typically measured, most often using a mean estimate of adherence to multiple refill dispensings associated with mean glycemic control calculated over a similar time period [5]. The shortcoming of this approach is that both adherence and A1C may vary substantially over time. To address this concern, we recently developed an adherence measure called the Biologic Response Based Proportion of Days Covered (BRB-PDC) that is calculated over the approximate 90-day period preceding an A1C measure and produced a stronger association between adherence and glycemic control than had been previously reported [6].

Although our BRB-PDC performed better than previous measures, it only relates glycemic control at a single point in time to adherence associated with that same point in time. To our knowledge, no study has evaluated whether* changes* in adherence correlate with* changes* in glycemic control. Therefore, we undertook the present study to determine whether initial adherence to metformin was associated with changes in HbA1c before and after metformin initiation and whether subsequent changes in adherence were associated with changes in glycemic control.

## 2. Methods

This was a retrospective cohort study of type 2 diabetes patients receiving medical care from the Kaiser Permanente Northwest (KPNW) integrated health system. KPNW provides comprehensive medical care to approximately 520,000 individuals within a 75-mile radius of Portland, Oregon. All medical utilization including inpatient admissions, outpatient visits, laboratory values, and pharmaceutical dispensings is captured in electronic medical records. The current observational study was reviewed and approved by the KPNW Institutional Review Board with a waiver of informed consent.

We identified patients with type 2 diabetes who initiated metformin as their first ever antihyperglycemic drug between January 1, 2007, and December 31, 2011. Inclusion criteria were at least one HbA1c measured within 6 months prior to metformin initiation and a second HbA1c measured between 6 and 12 months after metformin initiation (*n* = 3,109). We selected three subsets of these individuals to conduct three analyses of the relationship between adherence and change in A1C.

### 2.1. Adherence Measure

We calculated adherence using the Biologic Response Based Proportion of Days Covered (BRB-PDC) method. Briefly, this method estimates adherence over the 90 days preceding a given HbA1c measurement and allows multiple adherence measures in a defined time period (e.g., 1 year) rather than the traditional method that averages adherence and glycemic control over the given period. A detailed description of the BRB-PDC has been provided elsewhere and was shown to produce a stronger association between HbA1c and adherence than has been previously reported [6].

#### 2.1.1. Analysis 1

Our first analysis focused on the change in A1C prior to and 6–12 months following initiation of metformin among the 2,844 patients who had a 2nd metformin dispense any time after the first. Change in HbA1c was calculated as the first HbA1c 6–12 months after metformin initiation (Time 2) minus the last HbA1c on or before the date of the first metformin dispense (Time 1). Adherence was calculated using the BRB-PDC during the 90 days up to Time 2 and was categorized into 0%, 1–49%, 50–79%, or ≥80%. We modeled change in HbA1c as a function of HbA1c at Time 1, age, sex, BRB-PDC category at Time 2, and metformin dose at Time 2. The reference group was patients with no adherence (0%).

#### 2.1.2. Analyses 2 and 3

Of the 2,844 patients used in Analysis 1, we identified 2,418 patients who had at least one additional HbA1c measured after Time 2. We calculated change in HbA1c as of the last available HbA1c measure (Time 3) minus the first HbA1c 6–12 months after metformin initiation (Time 2). Adherence was calculated using the BRB-PDC at Time 2 and Time 3. Due to small cell size, we used three categories of BRB-PDC: no adherence (0%), some adherence (1–79%), or full adherence (≥80%). We modeled change in HbA1c as a function of HbA1c at Time 2, age, sex, change in BRB-PDC category from Time 2 to Time 3, and metformin dose at Time 3. Our third analysis was identical to the second but used only the 861 patients with HbA1c ≥ 7% at Time 2. The reference group for analyses 2 and 3 was patients whose categorized adherence did not change between Time 2 and Time 3. All analyses were performed with SAS, version 9.3 (SAS Institute, Cary, NC) using a variation of the conditional change regression model in a generalized linear model framework.

## 3. Results

The characteristics of the three samples were generally similar (Table 1). Patients not in glycemic control at Time 2 (Sample 3) were less likely to be newly diagnosed and to be receiving higher metformin doses at Time 3 compared with Sample 2. The distribution of categories of BRB-PDC was also similar for all three samples (Table 2). However, at Time 3 more patients had 0% adherence and fewer had full adherence in both Sample 2 and Sample 3.

**Figure 1:**
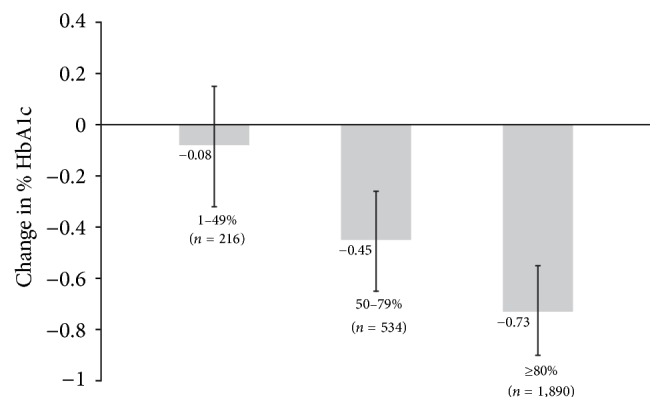
Percentage point change in HbA1c before and 6–12 months after metformin initiation by category of Biologic Response Based Proportion of Days Covered where 0% is the reference group (Analysis 1). Error bars represent 95% confidence intervals.

Figure 1 displays the relative change in HbA1c following metformin initiation using no adherence (BRB-PDC = 0%) as the reference category. Patients with BRB-PDC 1–49% did not have a statistically significantly different change in HbA1c. However, a BRB-PDC of 50–79% was associated with an adjusted reduction in HbA1c of 0.45 percentage points (−0.45; 95% CI −0.65, −0.26; *p* < 0.001) while BRB-PDC ≥ 80% was associated with an HbA1c change of −0.73% (−0.90, −0.55; *p* < 0.001). The model explained 64% of the variance in HbA1c change.

**Figure 2:**
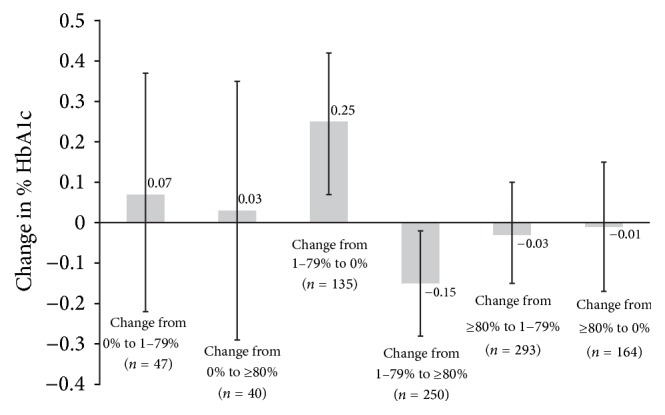
Percentage point change in HbA1c between first HbA1c measured 6–12 months after metformin initiation and last HbA1c of observation period measured 3–21 months later by change in category of Biologic Response Based Proportion of Days Covered where no change is the reference group. Data are for the total sample (Analysis 2). Error bars represent 95% confidence intervals.

**Figure 3:**
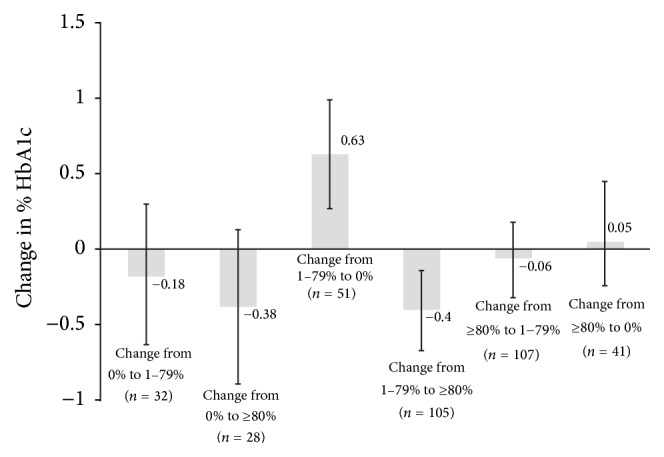
Percentage point change in HbA1c between first HbA1c measured 6–12 months after metformin initiation and last HbA1c of observation period measured 3–21 months later by change in category of Biologic Response Based Proportion of Days Covered where no change is the reference group. Data are for those whose HbA1c measured 6–12 months after metformin initiation was ≥7% (Analysis 3). Error bars represent 95% confidence intervals.

Figure 2 shows that among patients with at least some initial adherence (1–79%) at Time 2, change to total nonadherence (BRB-PDC = 0%) at Time 3 was associated with 0.25 percentage point increase in HbA1c (0.25, 0.07, and 0.42; *p* = 0.005) while change from some adherence (1–79%) to full adherence (≥80%) was associated with an HbA1c decrease of 0.15 percentage points (−0.15, −0.28, and −0.02; *p* = 0.027). Those associations were accentuated when limited to patients who were not in glycemic control at Time 2 (Figure 3). Change from some adherence (1–79%) to no adherence (0%) was associated with an HbA1c increase of 0.63 percentage points (0.63, 0.27, and 0.99; *p* < 0.001) and change from some (1–79%) to full adherence (≥80%) was associated with an HbA1c decrease of 0.40 percentage points (−0.40, −0.67, and −0.14; *p* = 0.003). However, performance of these models was poor, explaining only 10% and 7% of the variation in HbA1c change, respectively.

## 4. Discussion

In this observational study of approximately 3,000 patients, we found a strong association between adherence and reduction in HbA1c after newly initiating metformin therapy as their first ever antihyperglycemic drug; patients who were fully adherent experienced an HbA1c reduction of nearly three-quarters of a percentage point compared with patients who were not adherent. In addition, subsequent change in adherence (up to two years later) was also associated with change in HbA1c. Among patients who were initially partially adherent, the difference between becoming fully adherent and nonadherent was a full percentage point of HbA1c.

Many patients newly initiating metformin may be experiencing their first episode of chronic disease that will require daily medication. Our results indicate that* early* adherence has a profound effect on glycemic control. Better adherence is associated with better response to and durability of metformin monotherapy, as well as metformin and sulphonylurea combination therapy [2, 3, 7]. Because medication adherence represents a complex series of patient behaviors rather than a single construct [8], the cumulative glycemic burden experienced by diabetes patients over time could be substantially lowered by adherence behaviors established early in the course of diabetes.

To our knowledge, ours is the first study to examine the association between* change* in medication adherence and* change* in glycemic control. Within subgroups of change, we found a much stronger relationship between adherence and the 0.1%–0.3% of HbA1c reported in most previous studies of mean HbA1c and adherence [4] and stronger than our recent study that more directly linked adherence to the expected period of biologic response [6]. The present study suggests that, among patients not in glycemic control with some adherence (1–79%) at a given point in time, HbA1c can change by as much as 1 percentage point depending on whether these patients become completely nonadherent (increase in percent HbA1c of 0.63) or fully adherent (decrease in percent HbA1c of 0.40). We acknowledge that the performance of the statistical model producing these results was poor, and the sample size in many of the change categories was small. In addition, the reference category for the change models was “no change” in categories of adherence and the range of middle category (1–79%) was quite large, meaning that an individual could change their adherence by as much as 78% and still be counted as unchanged. This would bias the results towards the null, yet we nonetheless found some statistically significant results. Larger studies of change in adherence and change in A1C would allow for narrower adherence change categories and are needed to confirm our findings.

Our study has limitations to consider. KPNW is a large integrated health system with considerable information technology infrastructure, including a Panel Support Tool that overlays the EMR and alerts clinicians to care gaps, including elevated HbA1c [9]. As a result of this level of care management, our results may not be generalized to smaller practices or less sophisticated systems. However, these care management tools may also reduce the variation in HbA1c and adherence, which would make associations more difficult to find. As mentioned above, the small sample size in adherence change categories did not allow us to examine smaller changes in adherence that might be important. We also did not account for metformin intolerance, which may result in a different type of nonadherence behavior than that of patients who choose not to take their medication for other reasons. This is an important area for future adherence research.

## 5. Conclusion

Our study indicates that initial adherence to newly prescribed metformin therapy is a critical behavior that produces substantial HbA1c reduction. Subsequent change in adherence can also result in lower HbA1c, especially among those with modest adherence but suboptimal glycemic control. Whether such a patient becomes fully adherent or nonadherent was associated with a full percentage point difference in HbA1c, a benefit equivalent to initiating an antihyperglycemic agent [10].

## Figures and Tables

**Table 1:** Characteristics of three analysis samples.

	Sample 1 (*n* = 2,844)	Sample 2 (*n* = 2,416)	Sample 3 (*n* = 861)
Age	58.6	59.3	58.0
% men	54.1%	53.9%	57.7%
Nonwhite	18.1%	16.8%	20.7%
Duration < 1 year	55.9%	55.1%	44.4%
HbA1c at Time 1	8.3%	n/a	n/a
HbA1c at Time 2	7.0%	6.9%	7.8%
HbA1c at Time 3	n/a	7.1%	7.8%
Metformin dose (mgs) at Time 2	1,124	1,129	1,055
Metformin dose (mgs) at Time 3	n/a	1,223	1,404

**Table 2:** Distribution of Biologic Response Based Proportion of Days Covered (BRB-PDC) at Time 2 and Time 3 for each of the relevant study samples. Data are number (%) of patients.

	Sample 1 (*n* = 2,844)	Sample 2 (*n* = 2,416)	Sample 3 (*n* = 861)
BRB-PDC at Time 2			
0%	204 (7.2%)	105 (4.4%)	73 (8.4%)
1–49%	216 (7.6%)	177 (7.3%)	85 (9.9%)
50–79%	534 (18.8%)	445 (18.4%)	178 (20.7%)
>80%	1,890 (66.4)	1,689 (69.9%)	525 (61.0%)
BRB-PDC at Time 3			
0%	n/a	317 (13.1%)	105 (12.2%)
1–49%	n/a	145 (6.0%)	62 (7.2%)
50–79%	n/a	432 (17.9%)	184 (21.4%)
>80%	n/a	1,522 (63.0%)	510 (59.2%)
